# Supplementary material for: Nutrient enrichment induces dormancy and decreases diversity of active bacteria in salt marsh sediments
Source: Nat Commun. 2016 Sep 26;7:12881. doi: 10.1038/ncomms12881 (PMC5052679; doi:10.1038/ncomms12881)
Supplement: Supplementary Information — Supplementary Figures 1-3 and Supplementary Table 1 [file ncomms12881-s1.pdf]

Supplementary Information

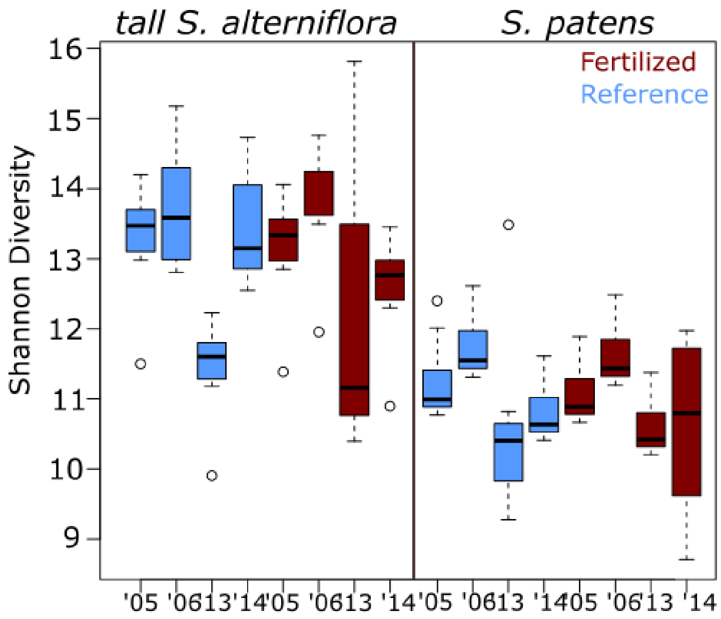

Supplementary Fig. 1. Nutrient enrichment did not alter diversity of the total microbial community. Box plots of average Shannon Diversity in the total community as assed by the 16S rRNA gene. Boxes are 25th and 75th quantiles with the medians in bold of samples collected each year from reference marshes (blue) and fertilized marshes (red). Although the total diversity of bacteria in *S. patens* is lower than that of *S. alterniflora*, there is no difference in diversity as a result of fertilization, in sharp contrast with what was observed in the diversity of the active portion of the community.

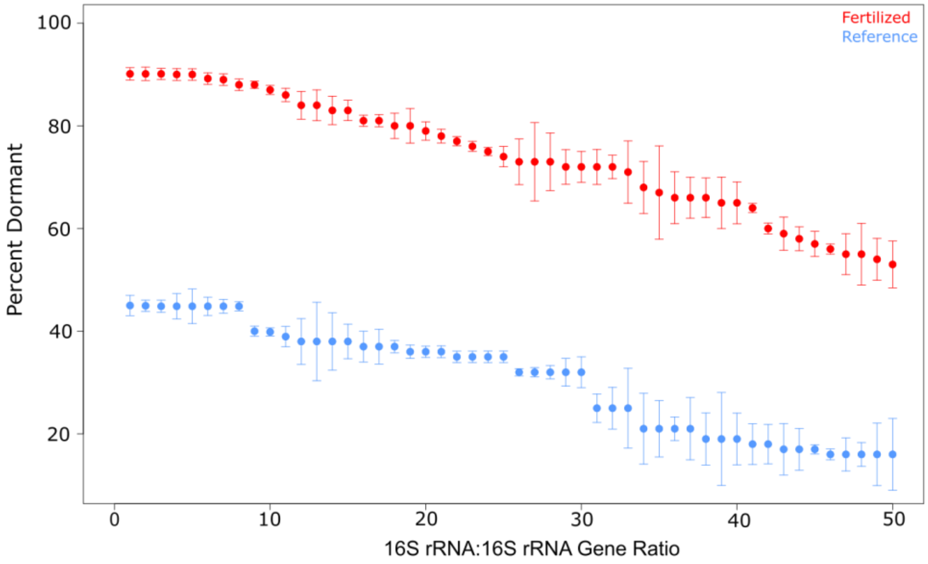

Supplementary Fig. 2. Regardless of how dormancy is defined, nutrient enriched marshes have consistently higher dormancy than reference marshes. Percent dormant taxa assessed by the ratio of 16S rRNA to the 16S rRNA gene. Data were generated by increasing the ratio required for a taxon to be considered active from greater than 1 to 50. At all ratios, fertilized creeks had significantly higher dormant taxa than reference sediments, as assessed by a one-way ANOVA.

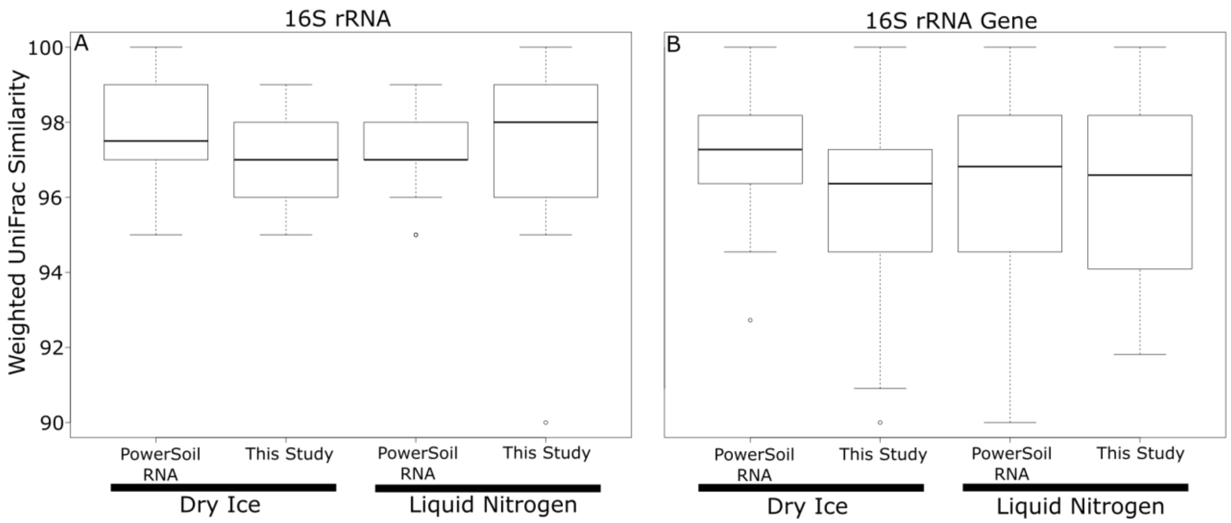

Supplementary Fig. 3. Comparison of nucleic acid preservation and extraction methods. Weighted UniFrac similarity comparing community similarity for 16S rRNA (A) and the 16S rRNA gene (B) for different extraction methods and sample preservation techniques.

Supplementary Table 1. **Significantly different microbial taxa.** Taxonomic information of the 32 significantly different (between fertilized and reference samples) OTUs present at least 100 times from figure 3 as determined by BLASTn. Bonferroni corrected p-values are significant at <0.001 as determined by a Kruskal-Wallis test.

	Phylum	Class	Order	Family	Bonferroni *P*
OTU1	Proteobacteria	Deltaproteobacteria	Desulfobacterales	NA	1.25E-14
OTU2	Cyanobacteria	Oscillatoriophycideae	Oscillatoriales	NA	6.51E-10
OTU3	Cyanobacteria	Oscillatoriophycideae	Oscillatoriales	NA	1.15E-02
OTU4	Cyanobacteria	Oscillatoriophycideae	Oscillatoriales	Phormidiaceae	1.68E-15
OTU5	Proteobacteria	Deltaproteobacteria	Desulfobacterales	Desulfobacteraceae	8.90E-13
OTU6	Proteobacteria	Deltaproteobacteria	Desulfobacterales	Desulfobacteraceae	4.87E-05
OTU7	Cyanobacteria	Oscillatoriophycideae	Oscillatoriales	NA	7.80E-11
OTU8	Cyanobacteria	Oscillatoriophycideae	Oscillatoriales	NA	3.34E-03
OTU9	Proteobacteria	Deltaproteobacteria	Desulfobacterales	Desulfobacteraceae	1.02E-06
OTU10	Proteobacteria	Deltaproteobacteria	Desulfobacterales	Desulfobacteraceae	1.50E-02
OTU11	Proteobacteria	Deltaproteobacteria	Desulfobacterales	Desulfobacteraceae	2.23E-09
OTU12	Cyanobacteria	Oscillatoriophycideae	Oscillatoriales	Phormidiaceae	2.47E-13
OTU13	Cyanobacteria	Oscillatoriophycideae	Oscillatoriales	NA	2.80E-14
OTU14	Proteobacteria	Deltaproteobacteria	Desulfobacterales	Desulfobacteraceae	4.26E-09

OTU15	Cyanobacteria	Oscillatoriophycideae	Oscillatoriales	NA	4.14E-10
OTU16	Proteobacteria	Deltaproteobacteria	Desulfarculales	Desulfarculaceae	7.03E-11
OTU17	Proteobacteria	Alphaproteobacteria	Rhodospirillales	Rhodospirillaceae	1.67E-09
OTU18	Cyanobacteria	Oscillatoriophycideae	Oscillatoriales	NA	1.28E-02
OTU19	Cyanobacteria	Oscillatoriophycideae	Oscillatoriales	NA	7.71E-14
OTU20	Proteobacteria	Alphaproteobacteria	Rhodobacterales	Rhodobacteraceae	2.75E-06
OTU21	Proteobacteria	Alphaproteobacteria	Rhodobacterales	Rhodobacteraceae	5.19E-09
OTU22	Proteobacteria	Alphaproteobacteria	Rhizobiales	Hyphomicrobiaceae	1.21E-08
OTU23	Proteobacteria	Alphaproteobacteria	Rhodospirillales	Rhodospirillaceae	2.11E-05
OTU24	Proteobacteria	Alphaproteobacteria	Rhodobacterales	Rhodobacteraceae	2.40E-06
OTU25	Proteobacteria	Deltaproteobacteria	Desulfarculales	Desulfarculaceae	1.73E-15
OTU26	Proteobacteria	Alphaproteobacteria	Rhizobiales	Hyphomicrobiaceae	8.47E-06
OTU27	Planctomycetes	Planctomycetia	Pirellulales	Pirellulaceae	7.47E-19
OTU28	Proteobacteria	Deltaproteobacteria	Desulfarculales	Desulfarculaceae	8.93E-17
OTU29	Proteobacteria	Alphaproteobacteria	Rhodobacterales	Rhodobacteraceae	3.46E-12
OTU30	Proteobacteria	Deltaproteobacteria	Desulfarculales	Desulfarculaceae	7.18E-14
OTU31	Proteobacteria	Alphaproteobacteria	Rhodobacterales	Rhodobacteraceae	7.79E-03
OTU32	Proteobacteria	Deltaproteobacteria	Desulfarculales	Desulfarculaceae	1.26E-02
